# Space Flight-Associated Neuroocular Syndrome, Idiopathic Intracranial Hypertension, and Pseudotumor Cerebri: Phenotypic Descriptions, Pathogenesis, and Hydrodynamics

**DOI:** 10.7759/cureus.14103

**Published:** 2021-03-25

**Authors:** Hassan Kesserwani

**Affiliations:** 1 Neurology, Flowers Medical Group, Dothan, USA

**Keywords:** idiopathic intracranial hypertension (iih), microgravity

## Abstract

Recent data from astronauts who have returned to Earth from a long-duration space flight have unequivocally distinguished spaceflight-associated neuro-ocular syndrome (SANS) from idiopathic intracranial hypertension (IIH) and pseudotumor cerebri (PTC). We review the semiology and pathogenesis of these three entities, noting that optic disc edema is what unites them, and this where the similarities between SANS and IIH/PTC end. We distinguish between PTC and IIH and between SANS and IIH/PTC and review the medical and surgical therapy of IIH/PTC. The key to understanding the phenomenon of optic disc edema is the geometry of the optic nerve sheath, which is a simulacrum of an inverted Venturi tube. This allows us to theoretically study the hydrodynamics of the optic nerve sheath by applying simple physical laws, including the Venturi effect, Poiseuille’s law, and Reynold’s number, and we speculate on nature’s design and the correlation of form and function in understanding how cerebrospinal fluid (CSF) circulates in the optic nerve sheath as it approaches the optic nerve head. Recent spectacular data on the histology of the blood nerve-barrier of the optic nerve disc and the glymphatic system of the optic nerve sheath will also help us understand the development of optic disc edema due to the microgravity-induced cephalad shift of CSF in SANS. We will explore the role of the sodium/potassium adenosine triphosphatase (ATPase) pump on choroid plexus epithelial cells and the aquaporin-4 water receptors located on astrocyte end-feet and their complex interactions with the tetracyclines, mineralocorticoids, and therapeutic agents with carbonic anhydrase activity. We also adumbrate the complex interactions between obesity, vitamin A, and 11-beta-hydroxysteroid dehydrogenase and how the aquaporin-4 receptor relates to these interactions.

## Introduction and background

We distinguish between spaceflight-induced neuroocular syndrome (SANS), idiopathic intracranial hypertension (IIH), and pseudotumor cerebri (PTC). The common denominator of SANS, IIH, and PTC is altered cerebrospinal fluid (CSF) hydrodynamics, the absence of a space-occupying lesion of the brain, the absence of ventriculomegaly, non-inflammatory CSF, and optic disc edema. SANS is due to altered CSF hydrodynamics induced by microgravity in outer space and its effect on the optic nerve disc; CSF pressure may be normal or mildly increased. PTC is due to raised CSF pressure induced by venous hypertension as seen with venous sinus thrombosis, an exogenous agent, such as hypovitaminosis A, hypervitaminosis A or tetracycline/minocycline, or other endocrine and neuro-humoral factors and their effects on the optic disc. IIH is also due to raised CSF pressure and occurs predominantly in younger obese women with no obvious immediate, proximate, endogenous, or exogenous etiology. Whether IIH/PTC is due to altered venous blood flow or altered CSF hydrodynamics or both is still debatable, with altered CSF hydrodynamics being the most likely cause. We will consider the terms CSF pressure and intracranial pressure (ICP) to be synonymous and apply them interchangeably. By hydrodynamics, we mean the pressure gradient across the lamina cribrosa of the optic disc, CSF pressure both intracranially and within the optic nerve sheath, and the dynamics of CSF flow along the optic nerve sheath. The distinguishing clinical features between IIH/PTC and SANS are listed in Table 1 [1].

**Table 1:** Distinguishing between IIH/PTC and SANS idiopathic intracranial hypertension (IIH), pseudotumor cerebri (PTC), spaceflight-associated neuroocular syndrome (SANS)

	TERRESTRIAL IIH/PTC	SANS
SEX	Ratio female-to-male is 9-to-1	Equal frequency
BODY HABITUS	Vast majority obese	Fit and athletic
HEADACHES	Frequent and severe	Occasional and rare
TRANSIENT VISUAL OBSCURATIONS	Frequent; almost three-quarter of patients	Never reported
DIPLOPIA	One-third of patients	Never reported
DISC EDEMA LATERALITY	Mostly bilateral	Mostly unilateral
CSF PRESSURE	Increased	Normal or mildly increased
CHOROIDAL FOLDS	Concentric around optic nerve head	Linear folds

Why SANS is mostly unilateral is not entirely clear, and the authors do not offer any explanation. It may have to do with local hydrodynamics and trabeculation/septation of the optic nerve sheath, as outlined in the Discussion section. Visual field testing in SANS revealed enlargement of the blind spot or scotomas. The nature of the scotomas was not identified in the report [1].

Walter Dandy published a series of 22 patients that had symptoms of high CSF pressure (headaches, transient visual obscurations, pulsatile tinnitus, diplopia), absence of tumor by ventriculography, and high CSF pressure with a lumbar puncture. All patients were decompressed by a subtemporal craniotomy with favorable long-term results. The absence of ventricular dilation suggested to him a lack of a CSF flow problem. He also made the astute observation that the chronicity of this condition precludes cerebral edema as a cause of this condition. The rapid relief with decompressive surgery was explained by invoking the Monroe-Kelley doctrine, that the drop in ICP reflects a drop in intracranial blood volume. He diagnosed these cases as " high intracranial pressure without a brain tumor," analogous to what we refer to now as pseudotumor cerebri [2].

The modified Dandy criteria are as follows: 1) a lumbar puncture confirming a high CSF pressure, greater than 200-250 mmHg in an adult and 280 mmHg in a child; 2) non-inflammatory CSF; 3) symptoms and signs concurrent with high CSF pressure with at least one of a) pulse-synchronous tinnitus, b) sixth nerve palsy, c) papilledema d) partially empty sella or distended or tortuous optic nerve sheath, e) magnetic resonance venous (MRV) with transverse venous sinus collapse or stenosis; 4) non-focal neurological signs except for a sixth nerve palsy; 5) no space-occupying lesion on magnetic resonance imaging (MRI) of the brain and no structural deformity of the ventricular system obstructing the flow of CSF; 6) funduscopic evidence of papilledema; 7) MRV imaging excluding a venous sinus thrombosis [3].

Friedmann DI et al. propose that these criteria be relaxed in patients with typical symptoms and signs of IIH/PTC but without papilledema especially in children and in adults early in the disease who may not register a high CSF pressure. This may be accounted for by subtle optic atrophy or fibrosis of the optic nerve fiber layer and may account for cases of obese women who have chronic daily persistent headaches [4].

The symptom profile of PTC/IIH reflects the complex hemodynamics of increased CSF pressure and its local effects (Table 2).

**Table 2:** The symptoms of IIH/PTC, their incidence, and the putative mechanism of action pseudotumor cerebri (PTC), idiopathic intracranial hypertension (IIH), intracranial pressure (ICP), cerebrospinal fluid (CSF)

	SYMPTOM	INCIDENCE (%)	MECHANISM
Weisberg LA [5]	Headache	99	Stretch of meninges, worse with supine posture (hydrostatic) and Valsalva maneuver
Giuseffi V, et al. [6]	Transient visual obscurations	68	Triggered by posture/transient ischemia of optic disc due to optic nerve swelling
Wall M [7]	Horizontal diplopia	38	Neuropraxia of the sixth cranial nerve due to high ICP and compression as it exits Dorello’s canal
Smith JL [8]	Pulsatile tinnitus	58	Resonance of arterial pulsations with venous pulsations due to venous hypertension or turbulent flow through stenosis of the transverse sinus. Improves with compression of ipsilateral internal jugular vein
Kleinschmidt KK, et al. [9]	Retrobulbar pain with eye motion	44	Due to swelling of optic nerve sheath
Friedmann DI, et al. [10]	Vision loss	30-35	See table 4
Digre KB, et al. [11]	Cervical radiculopathy	19	Increased CSF pressure in radicular nerve root sheath

Morphological changes induced by high CSF pressure on the eye globe (retrolaminar space), optic nerve sheath, and brain reflect both global and local hemodynamic changes (Table 3) [12-13].

**Table 3:** Morphological changes of high CSF pressure locally and globally on the eye-globe, optic nerve sheath, and brain cerebrospinal fluid (CSF), millimeter (mm)

MORPHOLOGICAL FINDING	MAXIMUM SENSITIVITY (%)	SPECIFICITY (%)
Empty sella	26.7	94.6
Slit-like ventricles	3.3	100
Enlarged optic nerve sheath	66.7	82.1
Bulging of the optic disc into eye-globe	30	100
Optic nerve enhancement	50	98.2
Tortuosity of the optic nerve	40	91.1
Flattening of lamina cribrosa	80	100
Transverse sinus narrowing	93	93
Cerebellar ectopia (more than 5 mm, peg-like)	21.9	Unknown

Papilledema reflects an increase in CSF pressure. Optic nerve swelling begins in the lower pole and progresses to the upper pole and then the nasal pole and reaches a climax in disc margin blurring and optic disc edema. There is concomitant venous distension and a loss of venous pulsation [14]. Interestingly, the papillomacular bundle is spared until late in the disease [15]. In late papilledema, there are retinal nerve fiber infarcts due to venous hypertension, leading to cotton-wool spots [16]. The effects of increased CSF pressure on the optic disc and retinal nerve fiber layer (RNFL) include (Table 4) [17].

**Table 4:** Effects of increased ICP on the optic disc and RNFL intracranial pressure (ICP), retinal nerve fiber layer (RNFL)

VISUAL FIELD DEFECT AND RNFL ABNORMALITIES	MECHANISM AND COMMENTARY
Arcuate-shaped	Mostly inferior periphery with more marked loss of nerve fibers in the superior retina (thinner layer superior optic disc, with less glial support)
Enlargement of the blind spot	Accumulation of peri-papillary sub-retinal fluid
Choroidal folds	Bulging of optic nerve sheath into eye-globe with crests and troughs of the choroidal layer or wrinkling of the retinal pigment epithelium
Peri-papillary hemorrhages	Leaky retinal arteries
Deep hemorrhages	Deep to retinal pigment epithelium; poor outcome
Star figure	Exudate: protein and lipid leakage from retinal capillaries between fovea and disc
Subretinal macular edema	10 % with severe papilledema
Subretinal neovascular membrane	Rapid vision loss

The mainstay of therapy is weight loss and the use of carbonic anhydrase inhibitors. The latter reduce CSF production and induce diuresis [18-19]. The older carbonic anhydrase inhibitor methazolamide was also effective. Though loop diuretics, bumetanide and furosemide, are less effective, they have some benefit [20]. Topiramate also works through its weight-loss action and carbonic anhydrase inhibition [21]. Further commentary on the details and recently discovered novel mechanisms of action of pharmacological agents will be outlined in the Discussion section.

Should medical therapy fail, surgical therapy falls into three categories: CSF diversion procedures, bariatric surgery, and venous stenting. A summary of the procedures and main principles and noteworthy points are listed below (Table 5).

**Table 5:** List of surgical procedures and their risk-benefit profile intracranial pressure (ICP), cerebrospinal fluid (CSF)

	CSF DIVERSION PROCEDURE OR OTHER SURGERY	COMMENT
Dandy DW [1]	Sub-cranial craniectomy (flap)	Dandy noted a rapid drop in ICP and ascribed it to arterial pressure and blood volume
Al-Saadany WF, et al. [22]	Lumbar-peritoneal shunt	Subarachnoid / 80 % success rate / frequent revision/lower infection rate than ventriculo-peritoneal shunt
McGirt MJ, et al. [23]	Ventriculoperitoneal shunt	Difficult placement as many patients have a slit-like ventricle but less shunt failure than lumboperitoneal shunts
Lee MC, et al. [24]	Cisterna magna shunt	When lumbar drainage and slit-like ventricles are not accessible
Banta JT, et al [25]	Optic nerve sheath fenestration	Slit in optic nerve sheath; may create a fistula or cut trabeculae allowing the release of retrolaminar pressure. Highly effective. Fenestration of one eye may also help the other eye via continuity of trabecular meshwork.
Johnston I, et al. [26]	Serial lumbar punctures	Short term: pregnancy, cryptococcal meningitis. Otherwise, high failure rate of 66%
Bussiere M, et al. [27]	Venous sinus stenting	High risk of complications and requirement of long-term dual antiplatelet therapy. Asymmetry in transverse sinuses as high as 73% in the general population. However, effective therapy
Sugerman HJ, et al. [28]	Bariatric surgery	Highly effective. Weight loss reduced CSF pressure to normal

SANS in a long-duration space flight leads to decreased near-vision; otherwise, astronauts are asymptomatic. Optic disc edema, eye-globe flattening, choroidal folds, and cotton-wool spots mimic what happens in terrestrial IIH. Globe flattening, which is the loss of curvature (convexity) of the posterior sclera, leads to the shortening of the globe and anterior displacement of the fovea, with a subsequent hyperopic shift of the refractive index. However, CSF pressure via lumbar puncture (LP) measurement is normal or mildly elevated post-flight. In microgravity, it is postulated that there is a cephalad shift in CSF leading to internal jugular vein distension and reduced CSF drainage into the venous system. This may lead to locally increased ICP and subsequent distension of the optic nerve sheath, stasis of axoplasmic flow, and disc edema. Surprisingly, despite the development of an empty sella, none of the astronauts developed any significant symptoms of increased ICP such as headaches, tinnitus, diplopia, or transient visual obscurations. The optic disc edema may last for six months post-mission [29]. Why the astronauts do not develop symptoms of increased ICP may be related to the fact that optic disc edema is due to local hydrodynamic changes at the optic nerve sheath and not global effects of increased ICP with stretching of the meninges and traction of the cranial nerves.

Interestingly, head-down tilting and prolonged bed rest can mimic microgravity-induced internal jugular venous distension and thickening of the retinal nerve fiber layer of the optic nerve on ocular computerized tomography (OCT), with thickening of the retinal fiber layer with OCT being an early marker of optic disc edema [30]. Increased ICP leads to elevated hypoxia-inducible factor 1-alfa (HIF-1 alfa) in the retinal ganglion cells (RGC) and leakage of the blood-optic nerve barrier with a spillover of fluid and protein in the optic nerve head and subretinal space in the papilla. Increased ICP may also lead to the increased stiffness of the trabecular meshwork in the optic nerve sheath increasing local CSF pressure [31-32]. The CSF may also become loculated within the optic nerve sheath explaining the persistent optic disc edema despite return to Earth in SANS and post-lumbar decompression in terrestrial IIH [33].

## Review

Anatomy of the optic nerve sheath and pressure differentials

**Figure 1:**
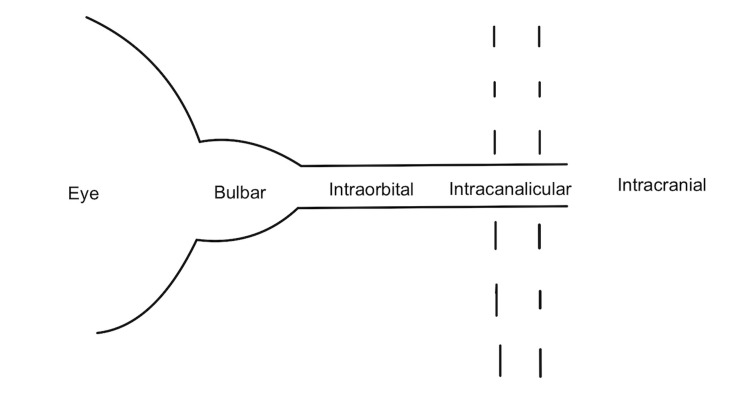
Optic nerve sheath subdivisions: bulbar, intraorbital, and canalicular segments

The optic nerve sheath is divided into three segments: the bulbar segment, the widest part, adjacent to the eye globe; and the intraorbital and intracanalicular segments. This tube is not necessarily a simple continuous tube with laminar flow (Figure 1).

Instead, between the pia and arachnoid layers, there is a fine trabeculated meshwork in the bulbar segment, loculation by septa and pillars in the intraorbital segment, and by trabeculae and pillars in the intracanalicular segment. This reticulated pattern is more like a complex web or a labyrinth where CSF reaches a dead-end at the eye-globe. CSF from the cisterns of the brain are pumped into this cavernous system almost like a hydraulic pump. Therefore, the optic disc is defended by the scleral lamina cribrosa, which is at the mercy of a pressure gradient between the intraocular pressure (IOP) and the ICP, where locally at the bulbar segment, the CSF pressure is referred to as the retrolaminar pressure (RLP). In glaucoma, IOP exceeds RLP and the optic disc is cupped into the retrobulbar space. When the RLP exceeds the IOP, the optic disc bulges into the eye-globe. Both scenarios alter the morphology of the lamina cribrosa, with compression leading to ischemia, retardation of axoplasmic flow, and neural degeneration of the retinal ganglion cells [34].

Hydrodynamics of the optic nerve sheath: Venturi effect, Poiseuille's law, and Reynold's number

**Figure 2:**
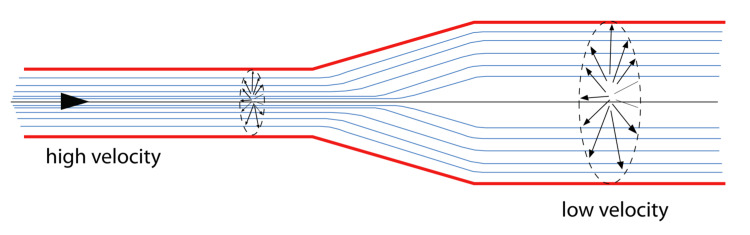
Reverse Venturi tube When moving from a lower cross-sectional area of the tubing to a higher cross-section area, the velocity of fluid flow decreases and the pressure head increases.

Why is the optic nerve sheath wider at the bulbar end? To answer this question, we need to return to the basics of hydrodynamics. When describing the flow of a fluid, we use the parameters of pressure $p$, density $\rho$, velocity $v$, and the volume flow per unit time $\frac{dV}{dt}$ across a cross-sectional area $A$. A Venturi pipe is one where the cross-sectional area decreases and a reverse Venturi pipe is one where the cross-sectional area increases as we travel along the pipe (Figure 2).

Assuming the net flow of fluid volume across any section of the tube is a constant, we have the simple differential equation:

$$\frac{dV}{dt}=\frac{A.v}{t}=constant$$

This tells us that the velocity of flow $v$ at any point along the tube is inversely proportional to the cross-sectional area $A$. Therefore, the velocity drops at the wider end of the reverse Venturi tube.

To explain what happens to the pressure $p$, we deploy Poiseuille's Law, which states: 

$$\frac{dV}{dt}=\frac{\pi pr^{4}}{8\eta l}$$

where $r$ is the radius of the tube, $l$ is the length, and $\eta$ is the viscosity of the fluid. There is an inverse relationship between the radius $r$ of the tube and the pressure $p$ at any point [35].

In summary, as we approach the bulbar end of the optic nerve sheaths, theoretically, two physical events happen, the CSF pressure increases and the speed of flow decreases. This actually follows from the Law of Conservation of Energy, where the total energy of a system (potential and kinetic energy) is conserved.

$$\frac{mp}{\rho }+\frac{mv^{2}}{2}=0$$

Again, we see that the pressure $p$ is inversely related to the velocity $v$ at any given point. This is nature's way of maintaining a pressure head when confronted with narrow bored tunnels as in the optic nerve sheath to allow CSF to bathe the optic nerve along its whole length.

How does one explain the septation and trabeculation of the optic nerve sheath? Here, we need to understand the concepts of laminar flow and turbulent flow. We believe that the septation and trabeculation of the optic nerve sheath induce turbulent flow when the ICP rises to high enough levels. This blunts the pressure head and protects the optic nerve sheath and head. To understand this, we need to know what Reynold's number is. Reynolds's number $R$ is the ratio of inertial resistance/viscous resistance expressed mathematically as:

$$R=\frac{\rho vl}{\eta }$$

**Figure 3:**
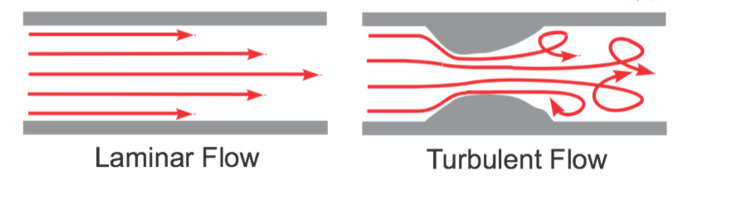
Laminar flow versus turbulent flow

When Reynold's number $R$ is low, the flow is steady, smooth, and laminar. When $R$ is high, the fluid flow is determined by the momentum more than the viscosity and the fluid flow is turbulent (Figure 3) [35].

**Figure 4:**
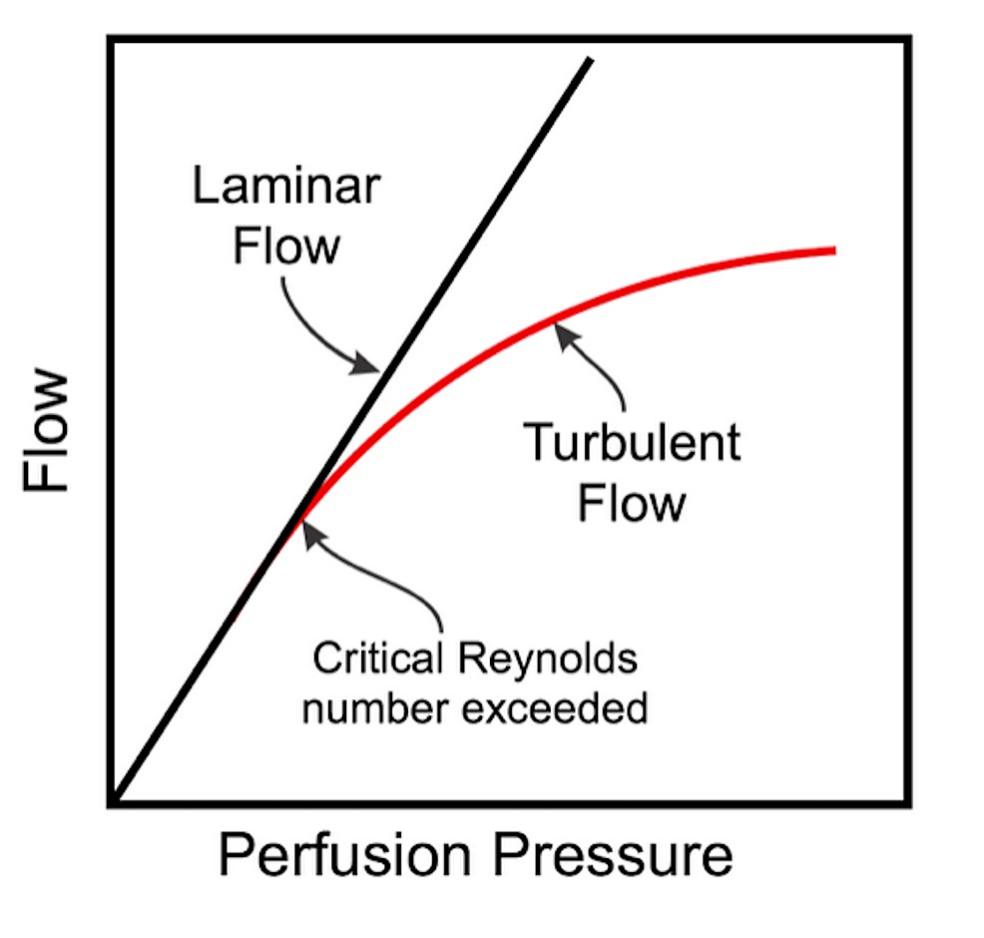
Flow-perfusion pressure diagram Note the drop in perfusion pressure when the flow is turbulent.

The septations and trabeculations create eddies and turbulent flow when the CSF pressure increases significantly, converting a laminar flow to a turbulent flow and reducing the pressure head at the optic nerve sheath (Figure 4).

These purely theoretical considerations are supported by experimental data. First, we know that CSF pressure determines the retrolaminar tissue pressure in the optic nerve sheath subarachnoid space based upon micropipette-pressure transducer recordings in an anesthetized dog [36]. Second, using microcomputerised scanning of porcine eyes, high CSF pressures correlated with deformation and distortion of the lamina cribrosa even with normal intraocular pressure [37]. Third, a remarkable study correlating optic nerve sheath diameter (a correlate of local CSF pressure) using transcranial Doppler and lumbar CSF pressure obtained a linear relationship between both parameters but with different slopes for different patients. The linear relationship occurred between 15 and 40 mmHg. After this, the optic nerve sheath reaches its maximum elasticity and yields, or based upon our hypothesis, the CSF flow reaches its Reynold's number and we produce turbulence and a drop in CSF pressure [38].

The role of aquaporins and a possible pathway for tetracycline pathology in PTC

CSF is produced by the choroid plexus (two-thirds of the volume) and the ependymal lining of the ventricles (one-third of the volume) with a gradual decline in the daily total volume with aging. Aquaporin-4 receptors on astrocyte end-feet facilitate the flow of water from the choroid plexus and ependymal epithelia into the CSF. There is also direct pumping of sodium into the CSF by the sodium/potassium adenosine triphosphates (ATPase) pump and the enzyme carbonic anhydrase that catalyzes the reaction between a carbon dioxide molecule and a water molecule to produce a proton and bicarbonate ions. The electrochemical gradient pushes protons and sodium ions along with water molecules into the CSF and drainage into the venous sinuses is across the arachnoid granulations and into the nasal lymphatics across the cribriform plate. The carbonic anhydrase activity inhibitors, acetazolamide, topiramate, and zonisamide have all been shown to inhibit aquaporin-4 activity. Nevertheless, the evidence for CSF overproduction in PTC/IIH is limited [39].

After injury or inflammation, activated astrocytes release matrix metalloproteinase-9 (MMP-9), which disrupts the basal lamina between the astrocyte end-feet and the endothelial cells. This leads to a breakdown in the blood-brain barrier and vasogenic edema facilitated by the upregulation of aquaporin-4 receptors. Adult rats subjected to traumatic brain injury developed less blood-brain barrier disruption with less upregulation of MMP-9 and aquaporin-4 receptors after injection with minocycline, an MMP-9 inhibitor. This was a beneficial effect but nevertheless provides evidence whereby the tetracyclines can modulate aquaporin-4 receptors and potentially lead to excessive CSF production [40]. Whether the tetracyclines act differently in non-injured brains and whether this is genetically determined is a complex question worthy of further study.

Obesity, inflammation, and 11-beta-hydroxysteroid dehydrogenase in IIH

Obesity and rapid weight gain in women are unequivocally very strongly associated with IIH, implying a major role for the neuroendocrine role of adiposity [41]. Even a less severe weight loss of 6% is a more potent catalyst for the resolution of papilledema than pharmacotherapy with acetazolamide [42]. Abdominal obesity may increase abdominal pressure and central venous pressure with secondary venous distension of the internal jugular vein and increased intracranial pressure. However, the association of CSF pressure (lumbar puncture) and body mass index (BMI) has a weak positive correlation. Obstructive sleep apnea increases ICP by increasing intrathoracic pressure and internal jugular vein distension and by hypoxia leading to hypercapnia with secondary vasodilatation.

Obesity is associated with elevated levels of serum and adipose 11-beta-hydroxysteroid dehydrogenase levels (11-beta-HSD). 11-beta-HSD regulates cortisol action intracellularly and increases the activity of the sodium/potassium ATPase pump, increasing the production of CSF in the choroid plexus epithelium. A fall in serum and CSF 11-beta-HSD levels correlated with a drop in CSF pressure and weight loss in a group of 25 obese patients with IIH [43]. There was a benefit of 11-beta-HSD inhibitors in a small phase two study of 31 obese women with IIH. The mean drop of CSF pressure was 4.3 cm water and the agent was safe and well-tolerated [44].

Obesity, polycystic ovarian syndrome (PCOS), vitamin A, and IIH

Adipose tissue contains the enzyme aromatase, which converts the androgen androstenedione to estrogens. Adipose tissue contains the enzyme aromatase, which converts the androgen androstenedione to estrogens. Estrogens are known to influence aquaporin-4 receptors and retinoic acid synthesis (see below) [45].

In IIH, the androgen profile is different from that in PCOS, despite the fact that PCOS is more prevalent in IIH than in the general population. In IIH, serum and CSF testosterone are increased and their precursors - dehydroepiandrosterone (DHEAS) and androstenedione - are decreased as compared to in PCOS and simple obesity. Testosterone is known to drive the choroidal plexus sodium/potassium ATPase, potentially increasing CSF output. The precursors DHEAS and androstenedione are the main circulating androgens in PCOS leading to hirsutism and android features, not seen in IIH [46].

Adipose tissue also increases the production of active vitamin A, retinoic acid, from the pro-hormone retinal. Both hypervitaminosis A and hypovitaminosis A can lead to IIH/PTC. Hypovitaminosis A can lead to fibrosis of the arachnoid villi in calves and impaired CSF absorption [47].

Microgravity-induced cephalad shift of fluid, local optic nerve sheath blood-nerve barrier defect, glymphatic flow, and SANS

SANS is a disease of the optic nerve head. As we outlined earlier, lumbar punctures performed post-long duration flight in astronauts have shown normal or mildly elevated CSF pressures. The cephalad shift of fluid in a microgravity environment leads to internal jugular vein distension and a puffy face. All astronauts develop optic nerve fiber layer thickening of the optic nerve head when visualized with OCT while 15 % of astronauts develop papilledema. Prolonged head-down tilt table posture can replicate these findings in the laboratory [48]. This pressure head facilitates the exudation of interstitial fluid in the pre-laminar region of the optic nerve head, where the blood-nerve barrier has been shown to be " leaky " as compared to elsewhere in the retina [49].

The glymphatic system is a paravascular channel pathway delimited by astroglial endfeet that allows intravitreal and retinal fluid to flow posteriorly into the optic nerve sheath subarachnoid space. The trans-laminar cribrosa pressure, which is equal to the intraocular pressure minus the ICP is about 4 mmHg. In microgravity, with the cephalad shift of CSF, this pressure differential is reversed and fluid is pressure-forced into the pre-laminar optic disc with the formation of optic disc edema. This idea is not far-fetched, as, in certain instances of subarachnoid hemorrhage, blood finds its way into the vitreous of the eye (Terson syndrome) [50].

## Conclusions

As spaceflight becomes increasingly common, the National Aeronautics Space Administration (NASA) has made SANS a top priority. Up to 15% of astronauts will develop optic disc edema and mitigating strategies, such as veno-occlusive thigh compression devices, are being investigated to counteract the cephalad shift of body fluids during space travel. As space tourists and travelers become commonplace, knowledge of SANS will become a prerequisite for the practicing clinician. In this article, we have distinguished between the clinical features and pathophysiology of SANS and IIH/PTC. We delve deeper into the hydrodynamics of the optic nerve sheath and the unique histological features of the lamina cribrosa and the glymphatic system, where we emphasized the role of translaminar pressure in the pathogenesis of optic disc edema. The complex interactions between obesity, vitamin A, the steroidogenic enzyme 11-beta-hydroxysteroid dehydrogenase, the aquaporin-4 receptor of astrocytes, and the sodium/potassium ATPase pump on choroid plexus epithelial cells and the generation of optic disc edema were explored. Finally, we draw attention to the non-traditional and novel mechanisms of action of the pharmacological agents used to treat PTC/IIH. This article is one of the earliest articles to address SANS in a non-space science-related journal.
